# Preparation of green high‐performance biomass‐derived hard carbon materials from bamboo powder waste

**DOI:** 10.1002/open.202300178

**Published:** 2024-01-12

**Authors:** Tianqi Yin, Zhengli Zhang, Lizhi Xu, Chuang Li, Dongdong Han

**Affiliations:** ^1^ Institute of Advanced Technology University of Science and Technology of China 230031 Hefei China; ^2^ CAS Key Laboratory of Urban Pollutant Conversion Department of Applied Chemistry University of Science and Technology of China 230041 Hefei China

**Keywords:** biomass, pyrolysis, biochar, hard carbon, sodium-ion battery

## Abstract

Efficient energy storage systems are crucial for the optimal utilization of renewable energy. Sodium‐ion batteries (SIBs) are considered potential substitutes for next‐generation low‐cost energy storage systems due to the low cost and abundance of sodium resources. However, the industrialization of SIBs faces a great challenge in terms of the anode. Hard carbon could be a promising anode material due to its high capacity and low cost which originates from biomass. This study used pre‐treatment and template carbonization methods to extract a hard carbon material from a large amount of discarded biomass in bamboo powder waste. This material has a good initial Coulombic efficiency of 78.6 % and good cycling stability when applied to sodium ion batteries.Typically, the optimal hard carbon material is used as the anode to prepare sodium ion battery prototypes to demonstrate their potential applications. The anode exhibited excellent sodium storage performance with a reversible capacity of 303 mAh ⋅ g^−1^ at 1 C rate and good cycling performance, retaining 92.0 % of its capacity after 100 cycles. These results demonstrate that BPPHC is a promising candidate for anode material in sodium‐ion batteries. This work suggests that bamboo powder could be a low‐cost anode material for SIBs.

## Graphical abstract

This study used pre‐treatment and template carbonization methods to extract a hard carbon material from a large amount of discarded biomass in bamboo powder waste. Template activation method produces rich and uniform pore structures that are beneficial for sodium storage.

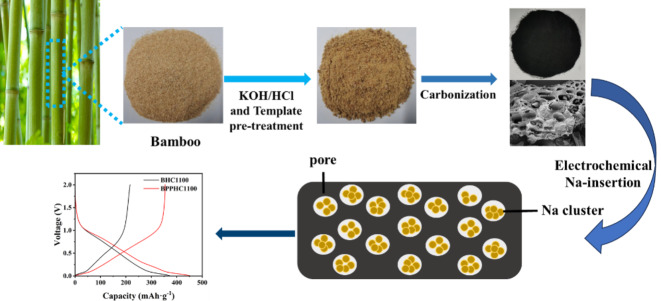

## Introduction

The demand for energy is increasing rapidly due to the sustainable development of modern industry and society. To meet this demand, various forms of sustainable energy, such as solar, wind, water, and biomass are being actively developed to meet the energy needs of human society. However, the development of efficient energy storage devices is becoming increasingly urgent to better utilize these energy sources. Over the past few decades, lithium‐ion batteries (LIBs) have become the primary power source for various energy storage devices due to their high energy density and long cycle life characteristics over the past few decades.[^1^, ^2^, ^3^, ^4^, ^5^] However, the limited availability of lithium resources and their uneven geographical distribution restrict their use in large‐scale energy storage. In contrast to lithium, sodium has similar intercalation chemistry as lithium and is one of the most abundant elements in the Earth's crust. As a result, sodium‐ion batteries (SIBs) are becoming the most promising alternative due to their lower cost, more widespread global reserves and higher storage capacity.[^6^, ^7^, ^8^, ^9^, ^10^, ^11^] Indeed, the performance of cathode and anode materials is crucial for energy storage devices. An excellent electrode material must satisfy several including low insertion potential, relatively stable structure, high electronic and ionic conductivity, and high compatibility with the electrolyte.[^7^, ^9^, ^12^, ^13^] Nevertheless, graphite, which is commonly used as an anode in lithium‐ion batteries, is no electrochemically inactive in sodium‐ion batteries. As a result, finding new anode materials with high sodium storage performance remains a significant challenge.[^14^, ^15^, ^16^]

Although various anode materials have been reported by researchers, such as carbon‐based materials, oxides and organic compounds, that exhibit a certain degree of sodium storage performance, most of these materials have demonstrated low capacity, poor cycle life, or safety issues due to high volume expansion during sodium insertion/extraction reactions.[^17^, ^18^, ^19^, ^20^, ^21^, ^22^, ^23^, ^24^, ^25^, ^26^, ^27^, ^28^] Research has shown that hard carbon materials are the most promising candidates for anode materials in sodium‐ion battery applications due to their larger interlayer distance, high capacity, excellent cycling stability, and lower operating potential.[^29^, ^30^, ^31^] In particular, the hard carbon derived from various biomass precursors, including peat moss, banana peels, corn cob, silk, wood cellulose, leaf and peanut shell, has gained significant attention and is currently under extensive research.[^32^, ^33^, ^34^, ^35^, ^36^, ^37^, ^38^, ^39^, ^40^, ^41^] However, the high cost of hard carbon materials, resulting in initial Coulombic efficiencies below 75 % and capacities below 300 mA ⋅ g^−1^ due to the formation of the solid electrolyte interface (SEI) layer, remains a major obstacle to their industrialization.^[38]^

This study reports the preparation of hard carbon materials from bamboo powder waste via a high‐temperature pyrolysis route and its use as anodes for SIBs.The electrochemical performance of the hard carbon derived from bamboo powder was found to be dependent on the carbonization temperature. The optimized hard carbon (carbonized at 1100 °C) exhibited stable cycling performance (a capacity retention of 92.0 % after 100 cycles) and high capability (363 mAh ⋅ g^−1^ at 0.2 C rate). Biochar with a high pore content can improve battery performance by facilitating electrolyte diffusion and Na ion transport, as well as expanding the interlayer spacing of graphite to de/intercalate Na ions, resulting in a higher stable reversible capacity. Furthermore, its highly specific surface area and excellent porous structure ensure its good sodium‐ion storage and cycling performance.

## Results and Discussion

**Figure 1:**
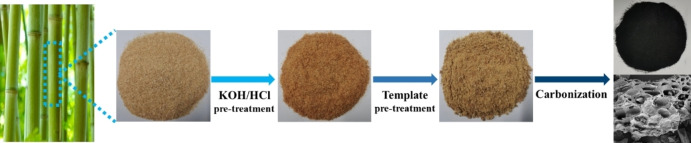
Schematic of the synthesis route and SEM image of BPPHC sample.

Bamboo powder waste was adopted as a precursor for hard carbon in this study, We designate unprocessed hard carbon materials as BHC, and those treated through the template method as BPPHC, and the synthesis process of hard carbon pyrolyzed from bamboo powder waste is shown in Figure 1. The bamboo powder turned black after high‐temperature carbonization, indicating a successful transition to a carbon material. The carbon yields from bamboo powder were measured at six different temperatures: 800 °C, 900 °C, 1000 °C, 1100 °C, 1200 °C, and 1300 °C (BHC800, BHC900, BHC1000, BHC1100, BHC1200, and BHC1300). The yields were 27.8 %, 25.7 %, 27.4 %, 29.0 %, 29.2 %, and 26.6 %, respectively. After the precursors are pretreated, the yields of hard carbon at 1000, 1100 and 1200 °C (BPPHC1000, BPPHC1100 and BPPHC1200) are 16.2 %, 16.6 % and 15.9 %, respectively.

**Figure 2:**
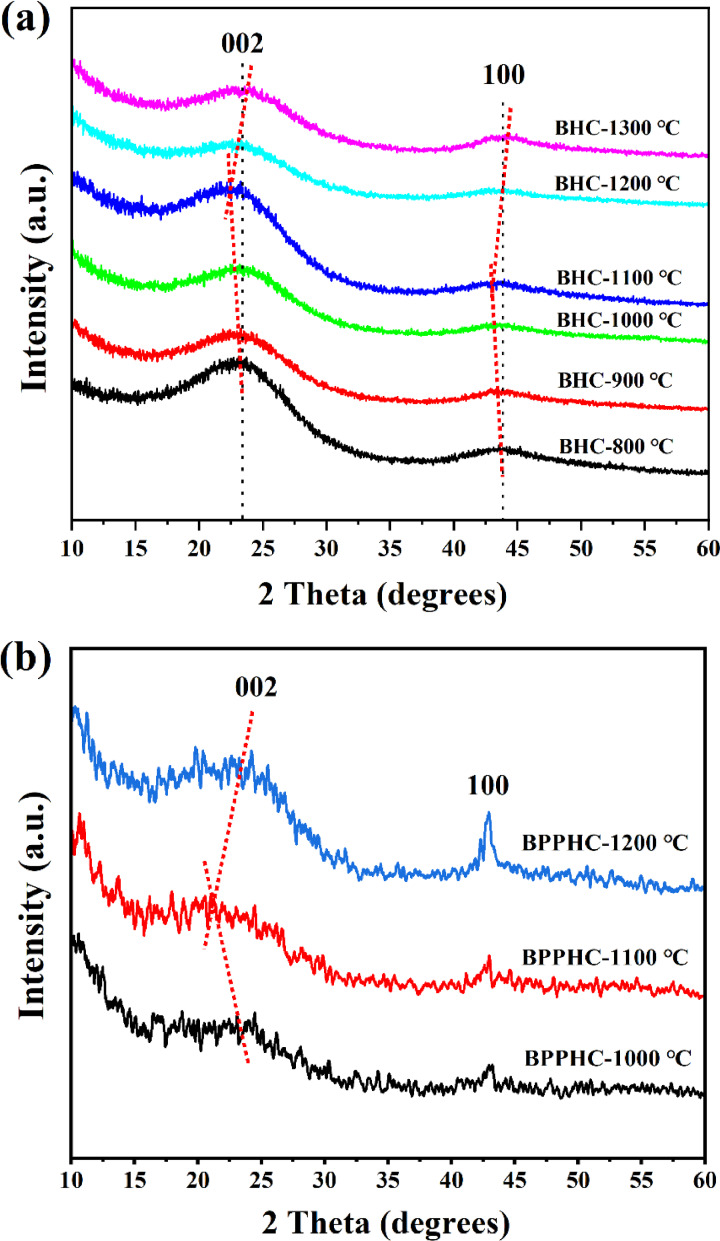
XRD patterns of different hard carbon samples: (a) XRD patterns of BHC at temperatures of 800, 900, 1000, 1100 and 1200 and (b) XRD patterns of BPPHC at temperatures of 1100, 1200 and 1300.

The XRD patterns for BHC800, BHC900, BHC1000, BHC1100, BHC1200 and BHC1300, which were synthesized from bamboo powder, were investigated to determine their crystallinities. The physical parameters estimated from the BET data were summarized in Table 1.The obtained black powders demonstrate non‐graphitizable carbon nature, as evidenced by the two broad peaks located at approximately 23° and 44° in 2θ, which are assigned to the (002) and (100) planes of graphite, respectively.[^42^, ^43^] From 800 °C to 1100 °C as the carbonization temperature increases, the (002) peak shifted to a lower angle and becomes narrower with increasing carbonization temperature, implying that the average graphene interlayer spacing (d_002_) increases and the thickness of graphitic domains (Lc) decreases.^[44]^ Whereas, further increasing the carbonization temperature from 1100 °C to 1200 °C, the (002) peak moved to a higher angle, implying that the average graphene interlayer spacing (d_002_) decreases, and it is speculated that a higher carbonization temperature will lead to the collapse of carbon layer structure.^[45]^ Fortunately, as the carbonization temperature increased, the (002) diffraction peak shifted but the peak pattern remained unchanged, indicating the hard carbon nature of BPPHC (Figure 2). The d_002_ values of the BPPHC1000, BPPHC1100 and BPPHC1200 are 0.456, 0.462 and 0.428 nm, respectively, as shown in Figure 2b. These values exhibit a dependence on the carbonization temperature. It also can be found that these hard carbons have a larger carbon layer spacing than that (0.335 nm) of graphite, which allows for facile Na^+^ insertion/extraction between the carbon planes.[^46^, ^47^]

**Table 1:** Physical parameters of the hard carbon samples synthesized by pyrolysis of bamboo powder.

Samples	BET/m^2^ ⋅ g^−1^	V_t_/cm^3^ ⋅ g^−1^	Pore Size/nm
BHC800	349	0.19	2.20
BHC900	356	0.20	2.19
BHC1000	447	0.25	2.26
BHC1100	486	0.27	2.19
BHC1200	415	0.24	2.34
BHC1300	224	0.13	2.29
BPPHC1000	420	0.68	6.95
BPPHC1100	1276	2.83	9.34
BPPHC1200	466	0.74	7.43

**Figure 3:**
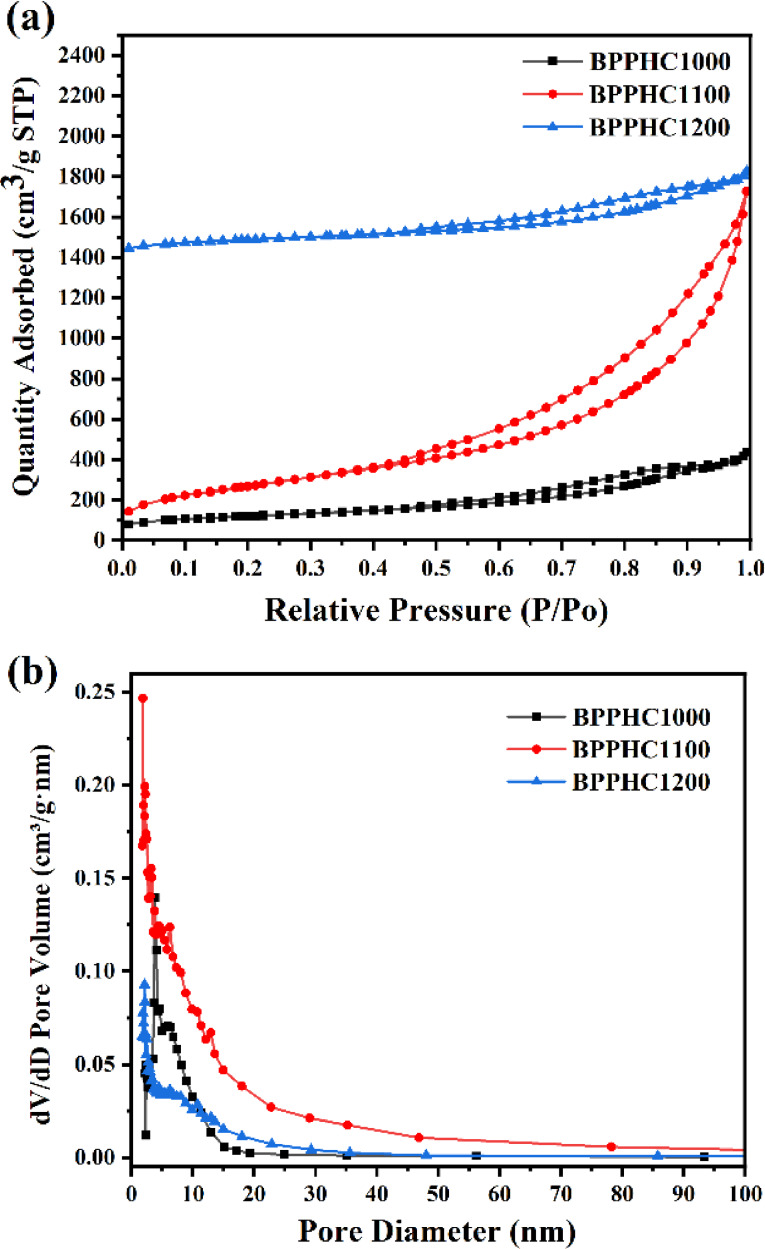
N_2_ adsorption‐desorption isothermal curves and the corresponding pore size distribution of the BPPHC carbonized at different temperatures. (a) N_2_ adsorption‐desorption isothermal curves of BHC at temperatures of 1000, 1100 and 1200. (b Pore size distribution diagrams of BPPHC at temperatures of 1000, 1100 and 1200.

The nitrogen adsorption/desorption method was used to determine the specific surface areas and pore size distributions of the hard carbon powders. The calculated values for BET surface area, total pore volume, and pore size are summarised in Table 1. As shown in Figure 3, the N_2_ adsorption isotherms of all samples have obvious desorption hysteresis loops. According to the classification of IUPAC, this is a typical type IV adsorption isotherm, indicating that there are a large number of mesoporous structures in the samples. The BET surface area of the BHC800, BHC900, BHC1000, BHC1100, BHC1200 and BHC1300 increased at first from 349 m^2^ ⋅ g^−1^ for the BHC800 to 486 m^2^ ⋅ g^−1^ for the BHC1100 and then decreased to 224 m^2^ ⋅ g^−1^ for the BHC1300. The trend can be explained that the increase of the surface area for the BHC1100 is mainly due to the removal of more functional groups and heteroatoms from the carbon structure to leave more nanovoids in the early stages of raising the carbonization temperature, and then the decrease of the surface area for the BHC1300 possibly results from the micropore closure or carbon layer structure collapse under excessively high temperature.^[48]^ Excitingly, the surface area and total pore volume of BPPHC samples increased significantly after pretreatment, and the surface area and total pore volume for the BPPHC1100 sample reached 1276 m^2^ ⋅ g^−1^ and 2.83 cm^3^ ⋅ g^−1^. The significant increase in the specific surface area and pore volume of the hard carbon material is mainly due to the increase of pore structure formation and carbon layer spacing caused by acid/alkali activation and template pretreatment. The Barrett‐Joyner‐Halenda (BJH) analysis revealed that the BPPHC 1100 sample (Figure 3a) exhibited type I isotherms, as classified by IUPAC. This indicates a higher surface area of the hard‐carbon material, with an average pore diameter of 9.3 nm, which is larger than that of BPPHC1000 (7.0 nm) and BPPHC1200 (7.4 nm) (Figure 3b). The existence of a large number of mesoporous structures in hard carbon materials can provide more structural space and facilitate the storage and migration of sodium ions.

**Figure 4:**
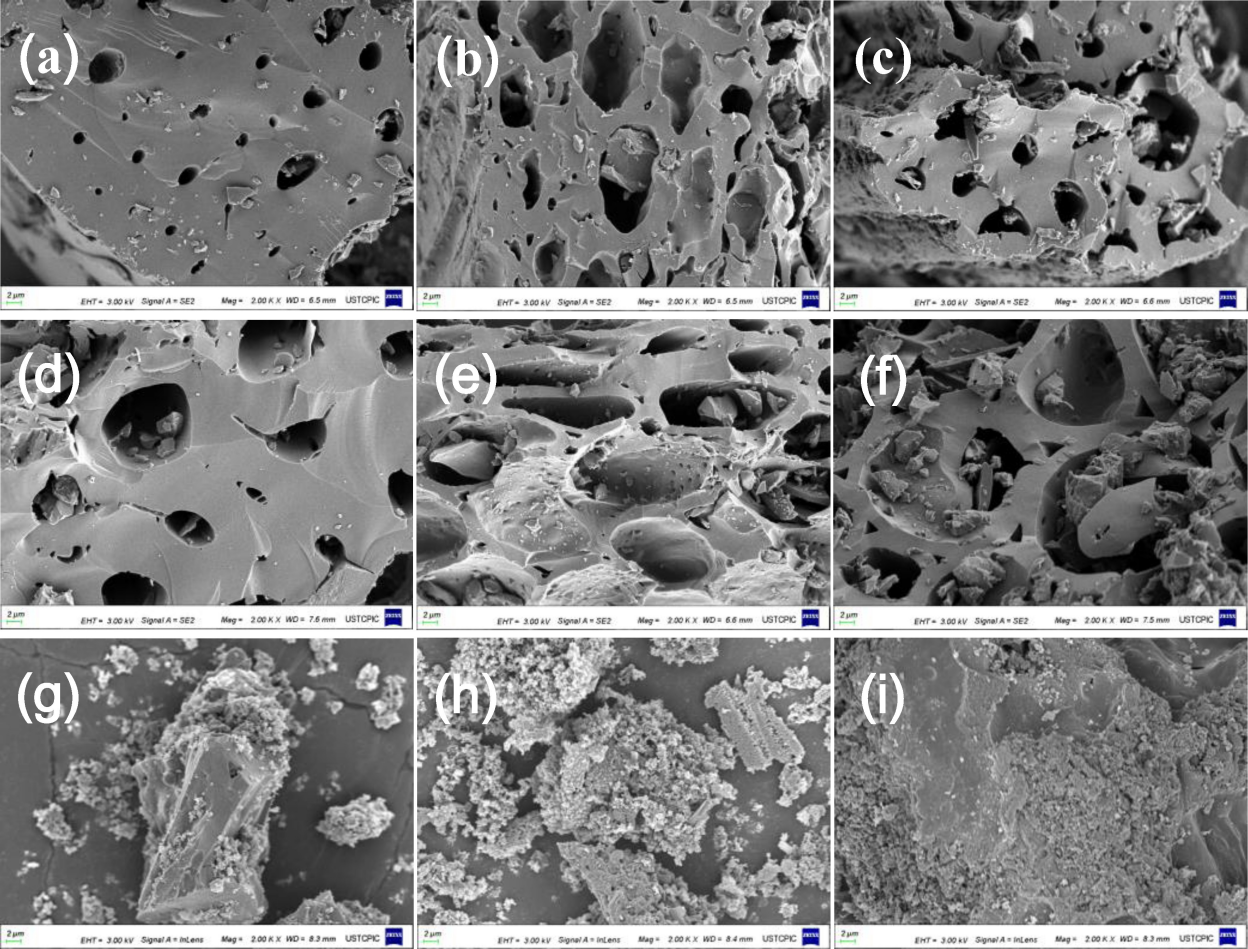
SEM images of (a) BHC800, (b) BHC900, (c) BHC1000, (d) BHC1100, (e) BHC1200, (f) BHC1300, (g) BPPHC1000, (h) BPPHC1100, (i) BPPHC1200.

**Figure 5:**
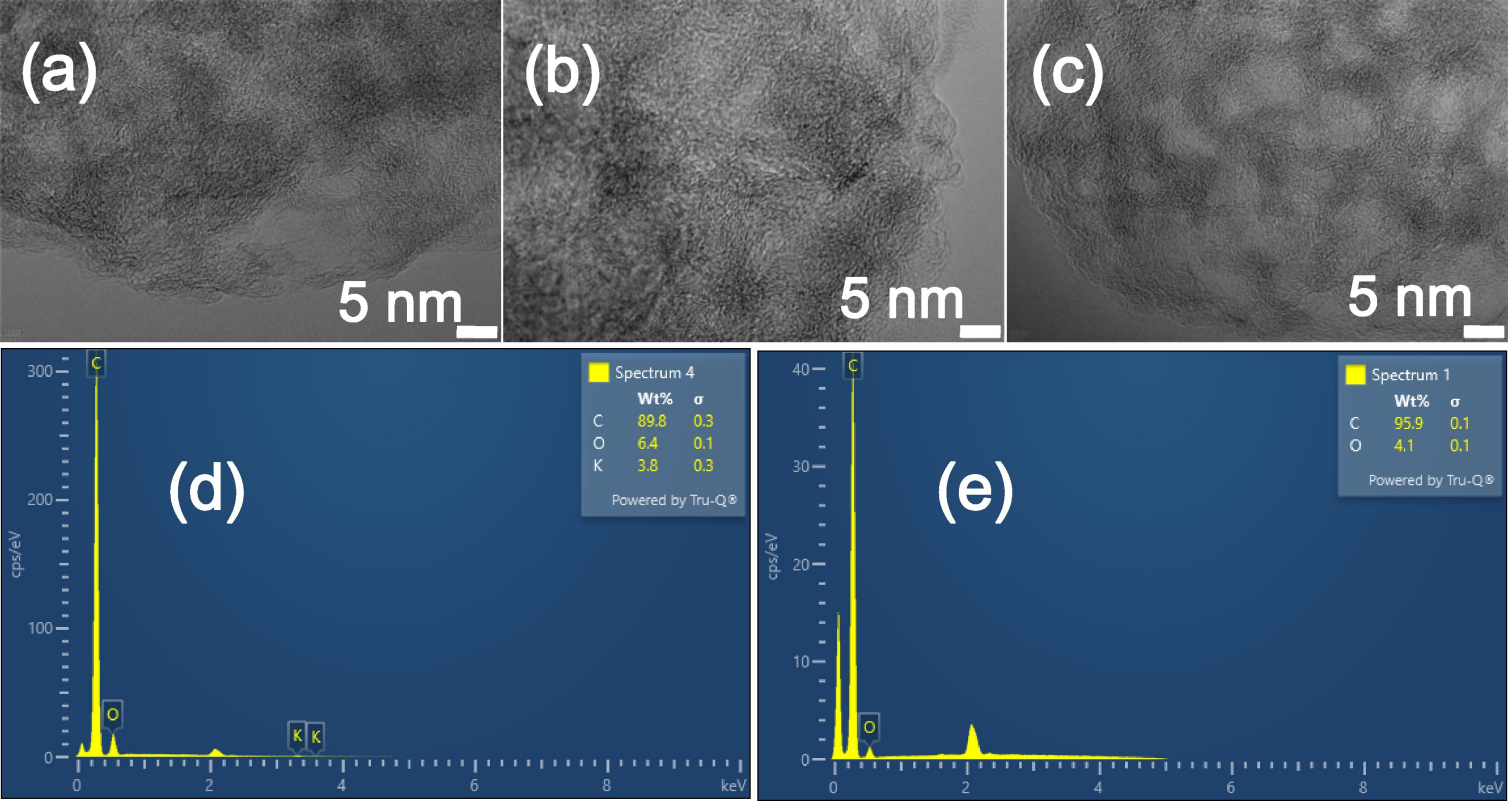
TEM images of (a) BPPHC1000, (b) BPPHC1100, (c) BPPHC1200. EDS images of (e) BHC1100, (f) BPPHC1100.

The microstructure and elemental composition of the BHC and BPPHC materials were further investigated using high‐resolution transmission electron microscopy (HRTEM), scanning electron microscope (SEM) and energy dispersive spectrometry (EDS). It can be observed from the SEM characterization results that natural pore structure is formed after bamboo powder carbonization and the pore structure size increases with the increase of carbonization temperature, and the formation of a large number of pore structure has a huge advantage on the storage of sodium ions, as shown in Figure 4a–e. However, when the carbonization temperature reaches 1200 °C, the carbon material structure collapses to a certain extent (Figure 4f). Figure 5a–c (HRTEM) shows that the BPPHC samples do not have prominent long‐range structural ordering. Instead, distinct turbostratic graphitic microstructures are observed, which exhibit a feature of hard carbon materials. However, they show different shortrange structures. The graphite‐like micro‐crystallites constructed by several short‐range parallel carbon hexagonal layers increase evidently with the increase in carbonization temperature, which is due to rearrangement of carbon atoms. The collapse of the microstructure results in the formation of numerous closed nanovoids in BHC samples subjected to higher heat treatment temperatures, thereby creating additional sodium storage sites.

Based on the XRD results, we calculated an expanded interlayer spacing for BPPHC, facilitating the intercalation of sodium ions and thereby increasing the capacity of the intercalation platform. The SEM and TEM images clearly reveal an enhanced porous structure in the BPPHC material, characterized by abundant nanoscale pores. Moreover, a higher removal of carbon atoms is observed, favoring increased sodium ion embedding. The XRD, HRTEM, and EDS analyses reveal a highly disordered structure, which facilitates the storage and transmission of Na+ ions. This makes it a suitable anode material for SIBs. This is beneficial for maintaining battery performance. Obviously, there is some ash in the carbonized raw bamboo powder, and the carbonized bamboo powder after the acid‐base pretreatment process shows a relatively pure carbon component. as shown in Figure 5j–k, which avoiding damage to battery performance.

**Figure 6:**
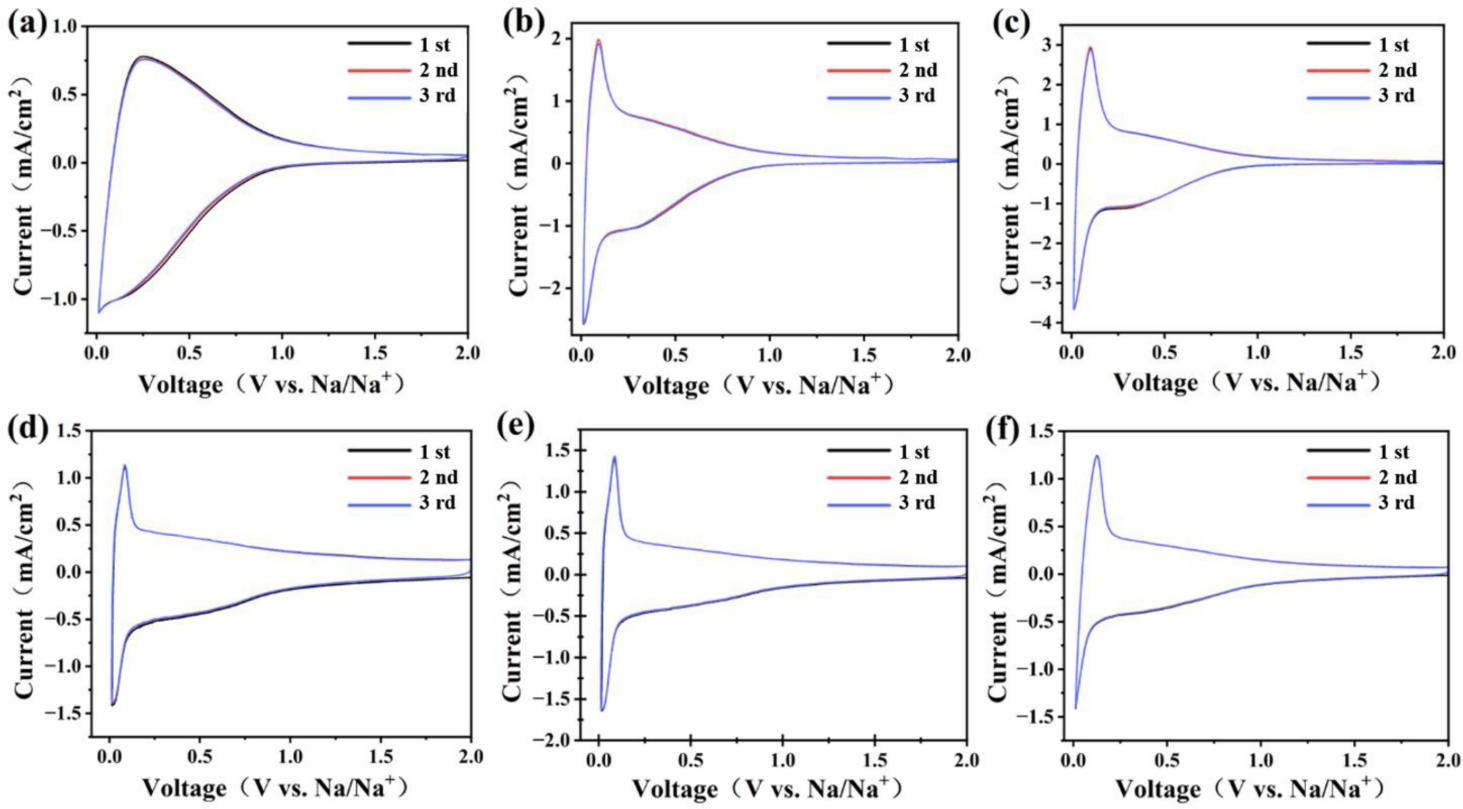
Cyclic voltammetry (CV) curves of (a) BHC1000, (b) BHC1100, (c) BHC1200, (d) BPPHC1000, (e) BPPHC1100 and (f) BPPHC1200.

In order to investigate the sodium storage performance of hard carbon materials, sodium‐ion batteries assembled with hard carbon negative electrode materials were subjected to CV testing in the potential range of 0.01–2 V at a scan rate of 1 mV ⋅ s^−1^.[^46^, ^49^, ^50^] The results showed that the discharge/charge curves of the hard carbon material samples prepared at different carbonization temperatures exhibited similar shapes, including a high potential slope region above 0.1 V and a low potential plateau region below 0.1 V (Figure 6). corresponding to different sodium storage mechanisms. The results indicate that the charge and discharge curves of the hard carbon material samples prepared at different carbonization temperatures have a consistent basic shape, with a high potential slope region above 0.1 V and a low potential plateau region below 0.1 V, corresponding to different sodium storage mechanisms. The high voltage slope area corresponds to the sodium ion storage on the surface sites, while the low voltage slope area corresponds to the insertion of sodium ions between the carbon layers. The highly overlapping CV curves from three scans confirm the reversible insertion and extraction of Na^+^ ions in the hard carbon structure, indicating excellent stability and reversibility of the prepared hard carbon anode material for Na^+^ ion storage.

The theoretical coulombic efficiency of the material can reach a high level. For the BHC sample, the high voltage sloping region shows lower capacity storage, typically for the BHC1000 where the capacity is mainly on the low voltage platform due to the fewer surface sites resulting from the lack of defects and pore structures in the carbon layer. BHC1100 and BHC1200 samples show a reduction peak at 0.3 V in the CV curves, which may be attributed to the generation of solid‐state electrolyte SEI membrane. The CV curve of the BPPHC sample shows a pair of sharp oxidation‐reduction peaks, which have similar shapes and peak positions with a small polarization, indicating a strong electrochemical reversibility. After acid/base and template treatment, the BPPHC sample showed strong reversibility, indicating that BPPHC hard carbon material was easier to absorb Na^+^ ion and has a faster charge transfer rate, which is attributed to the larger carbon interlayer spacing and more pore structures in BPPHC material. As for the BPPHC1100 hard carbon material, the increased capacity in the high‐voltage sloping region of the CV curve can be attributed to an increased specific surface area after pretreatment (increased from the original 486 m^2^/g to 1276 m^2^/g), a more abundant pore structure, a significantly increased pore volume (from 0.27 V/cm^3^ to 2.83 V/cm^3^) and a noticeable increase in average pore size (from 2.19 nm to 9.34 nm).

Furthermore, the results confirm that the pre‐treated BPPHC carbon material shows higher capacity storage in both the low voltage platform and high voltage sloping regions, and the abundant pore structure of the hard carbon material is beneficial for sodium ion storage. The CV characterization further confirmed that the pretreated BPPHC carbon material exhibited higher capacity storage in both the low‐voltage plateau region and the high‐voltage sloping region, and the rich pore structure of hard carbon material contributes to the storage of sodium ions.

**Figure 7:**
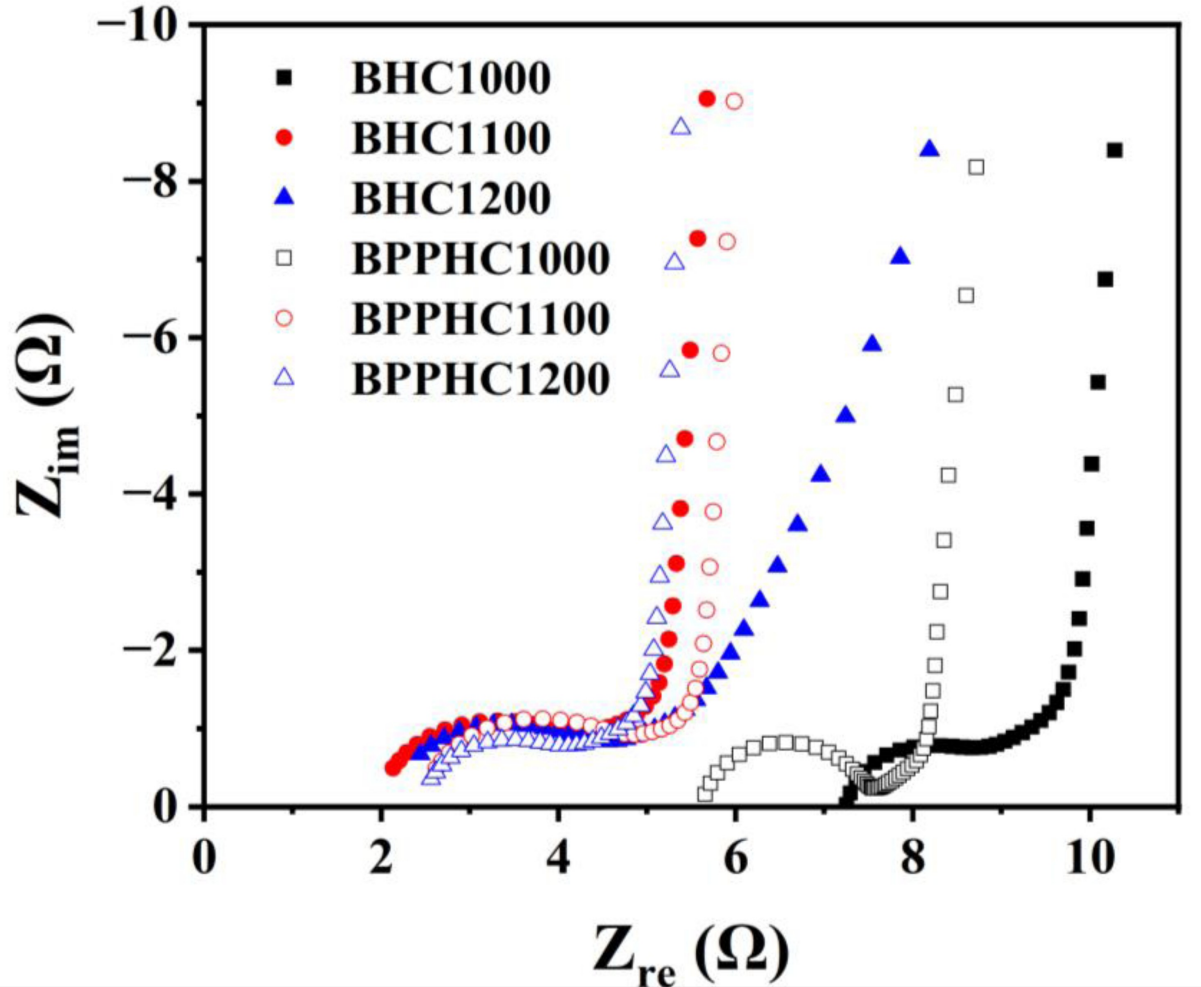
Nyquist impedance data of BHC electrodes at temperatures of 1000, 1100 and 1200 and BPPHC electrodes at temperatures of 1000, 1100 and 1200.

The impedance performance characterization of the hard carbon material showed an arc‐shaped charge transfer curve instead of the expected semicircle. This is likely due to the unevenness of the electrode surface, poor conductivity of the adsorption layer, and SEI film. The circular diameter of the BHC1000 hard carbon material was the smallest, indicating that its charge transfer resistance was the lowest. As the temperature rises, the charge transfer resistance of the hard carbon material increases. For the BHC1200 sample, the smaller slope of the low‐frequency region line indicated that the migration resistance of sodium ions inside the material was greater. For the BPPHC sample, as the temperature increased, the circular diameter of the hard carbon material increased, indicating that its charge transfer resistance was increasing. The impedance of charge transfer resistance is closely related to the pore distribution of the hard carbon material. From the Figure 7, it can be observed that the Weber impedance of the BPPHC sample was smaller, indicating that the pore size distribution range of the treated sample was wider and had abundant mesopores. The mesopores act as a channel for the migration of sodium ions, which can enable electrolyte ions to quickly diffuse into the pore of the hard carbon material.

**Figure 8:**
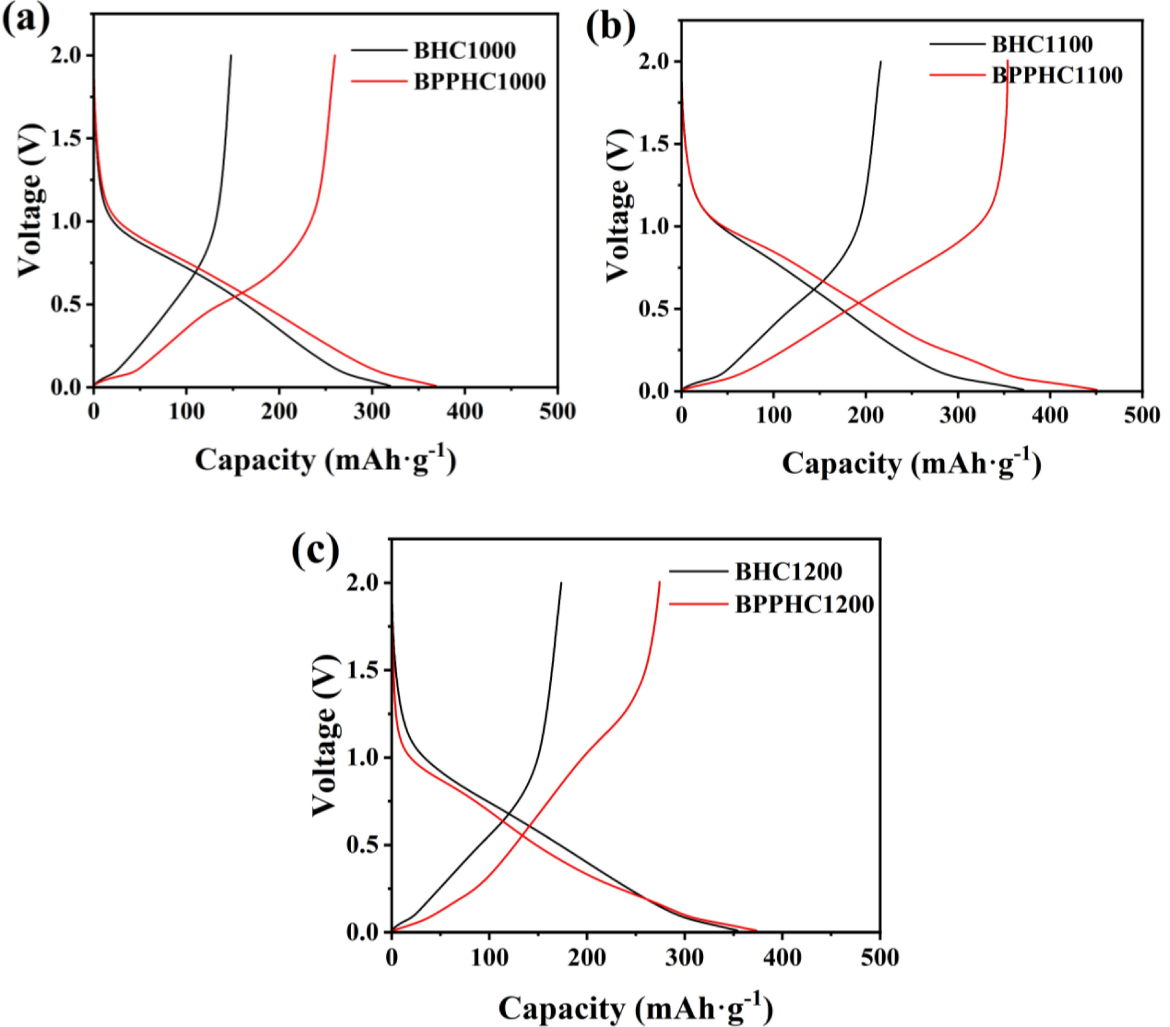
The first discharge/charge profiles of(a) BHC and BPPHC at temperatures of 1000 (b) BHC and BPPHC at temperatures of 1100 (c) BHC and BPPHC at temperatures of 1200.

The initial galvanostatic discharge/charge curves for different BHC electrodes tested at a current rate of 0.2 C (50 mA ⋅ g^−1^) within the potential range of 0~1.2 V (vs Na^+^/Na) are shown in Figure 8. The initial discharge capacities of BHC1000, BHC1100, and BHC1200 electrodes were 319 mAh ⋅ g^−1^, 371 mAh ⋅ g^−1^ and 354 mAh ⋅ g^−1^, respectively, and the initial coulombic efficiencies were 47.7 %, 58.7 % and 50.4 %, respectively. As shown in Figure 8, the initial discharge capacities of BPPHC1000, BPPHC1100 and BPPHC1200 electrodes were 368 mAh ⋅ g^−1^, 450 mAh ⋅ g^−1^ and 373 mAh ⋅ g^−1^, respectively. With the increase of carbonization temperature, the initial coulombic efficiency increased to 78.6 % at 1100 °C. However, further increasing the carbonization temperature to 1200 °C resulted in a decrease in initial coulombic efficiency to 73.4 %, This is mainly because the pore structure of the pre‐treated carbon material was significantly increased, with specific surface area increasing from 420 m^2^ ⋅ g^−1^ to 1276 m^2^ ⋅ g^−1^, and total pore volume and pore size also increased. After the carbonization temperature was increased to 1200 °C, the specific surface area, total pore volume and pore size of the carbon material decreased, which may be due to the excessively high carbonization temperature causing the closure of micropores or the destruction of carbon layer structure. In addition, reversible capacity loss may be caused by the electrolyte partially decomposing on the surface of the active sites, and partially decomposing on the carbon surface to form a solid electrolyte interphase (SEI) film. The above results indicate that the initial coulombic efficiency of the hard carbon material significantly increased after acid/base and template pretreatment, and the initial coulombic efficiency of the BPPHC1100 sample reached 78.6 %, while the initial coulombic efficiency of the BHC1100 sample was low, possibly due to the high content of oxygen‐containing functional groups on the surface of the untreated hard carbon material, which made it difficult for sodium ions to be desorbed. Following pretreatment, the bamboo biomass raw material exhibited a decrease in surface oxygen‐containing functional groups, resulting in an increase in initial coulombic efficiency. After pretreatment and carbonization at 1100 °C, the specific surface area, porosity, pore volume and degree of disorder of the hard carbon material were significantly improved.

After secondary treatment, the specific surface area and pore volume of the BPPHC samples significantly increased. Porous structures were formed and the interlayer spacing of carbon was expanded due to acid/base activation and template pre‐treatment. Due to the filling of the soft and hard template agents in the internal pores of bamboo powder, during high‐temperature carbonization, (CH_3_COO)_2_Mg ⋅ 4H_2_O generated MgO attached to the surface of the hard carbon material. The BPPHC1100 hard carbon material served a dual purpose of creating and preventing pore collapse. The specific surface area and total pore volume of the BPPHC1100 hard carbon material reached 1276 m^2^ ⋅ g^−1^ and 2.83 cm^3^ ⋅ g^−1^, respectively. The average pore diameters of the BPPHC1000, BPPHC1100, and BPPHC1200 hard carbon materials. These factors collectively contribute to the excellent electrochemical performance of the BPPHC1100 sample. Therefore, the above characterization results confirm that the BPPHC1100 sample is relatively suitable for use as a negative electrode material in sodium‐ion batteries.

**Figure 9:**
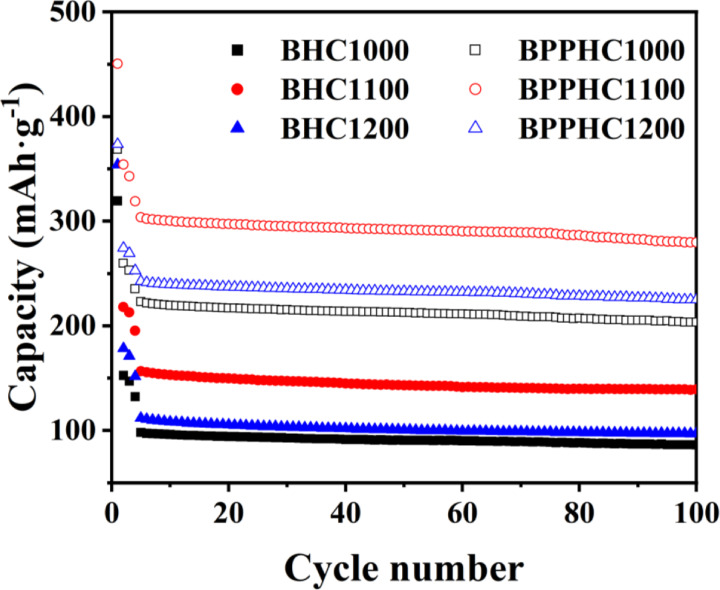
Cyclic performance of BHC and BPPHC at temperatures of 1000, 1100 and 1200 under a current rate of 1 C.

The cycling life of electrode materials is an important indicator of battery performance requirements, which determines whether they have the potential for commercial applications. Figure 9 shows the constant current charge‐discharge cycling capacity of the hard carbon electrode material, it can be observed that the hard carbon material exhibited lower coulombic efficiency in the first 5 cycles, which mainly due to the fact that the porous structure of the sample has a larger specific surface area, allowing for greater contact with the electrolyte and resulting in a larger SEI film formation area and consumption of more sodium ions. The material also contains a large number of microporous structures inside, making it difficult for the electrolyte to penetrate and causing sodium ions to easily undergo irreversible adsorption. Additionally, irreversible adsorption of oxygen‐containing groups on the surface of the material can cause the loss of sodium ions. Compared to the BHC sample, the BPPHC sample after pretreatment showed a significant improvement in coulombic efficiency. After 5 cycles, both the BHC and BPPHC samples reached a stable state, with the reversible sodium storage capacity of the BPPHC1100 sample maintained at 303 mAh ⋅ g^−1^. After 100 cycles, the capacity retention rate of the BPPHC sample remained above 92.0 %. The characterization results of the cycle life of electrode materials confirmed that the hard carbon material prepared from bamboo biomass raw materials after pretreatment has excellent cycling stability performance.

## Conclusions

This work successfully synthesised a hard carbon material of BPPHC derived entirely from a single precursor of biomass waste bamboo powder with a convenient, economical and scalable method were successfully synthesized. The study deeply investigated key factors of pre‐treatment techniques and carbonization temperatures on the carbon structure and electrochemical performance. When evaluated as a potential anode for SIBs, the biomass‐derived hard carbon material derived from biomass known as BPPHC1100 demonstrated a high reversible capacity (303 mAh ⋅ g^−1^ at a rate of 1 C) and superior cycling performance (a capacity retention of 92.0 % after 100 cycles). The material contains numerous nanoscale pores, which are formed through acid/base activation and template pre‐treatment, resulting in the expansion of the interlayer spacing of carbon. The material benefits from both soft and hard templates, which create pores at specific sites and prevents pore collapse. The templates have a dual role in pore formation and stability: they occupy sites for pore creation and mitigate pore collapse. This dual role of soft and hard templates contributes to the enhancement of pore formation and stability in the material. This study provides valuable insights into the mechanisms underlying the rich nanoporosity of the material. This contribution presents a method for producing hard carbon materials from waste bamboo powder waste and low‐cost anode materials in SIBs for large‐scale energy storage applications.

## Experimental Section

### Pretreatment of bamboo powder waste

Fujian Furen Group Forest Industry Company in Fujian Province provides bamboo powder. Initially, bamboo materials are crushed to a particle size of 30 mesh and subsequently cleaned and dried using deionized water. Factors that may influence bamboo powder waste include growth cycles, bamboo sections, and species, among other. 20 g of initial bamboo powder waste was dissolved in 200 mL of KOH aqueous solution (3 mol/L) and stirred for 24 h. The resulting sample was filtered and washed with deionized water until pH was adjusted to 7.0. The bamboo powder of alkali treatment was eventually obtained after being filtered, washed and dried at 105 °C. The bamboo powder that underwent alkali treatment was dissolved in 200 mL of HCl aqueous solution (1 mol/L) and stirred for 8 h. Then sample was filtered and washed with deionized water until pH was adjusted to 7.0. The the bamboo powder that underwent acid treatment was eventually obtained after being filtered, washed and dried at 105 °C.

### Materials synthesis of BPPHC

The bamboo powder, which was pretreated as described above, and Pluronic F127 were dissolved in 200 ml of THF. An additional 1.34 mL of 6 M HCl was added to the solution. Simultaneously, Mg(CH_3_COO)_2_ ⋅ 4H_2_O was simultaneously dissolved in 300 mL of H_2_O, and slowly poured into the previous solution. Afterward, the mixed solution was stirred at room temperature for 24 hours, then left at 45 °C for 1 day and held at 70 °C until the overall mass was thoroughly dried. Then the dired mixture mass was carbonized in a tube furnace under nitrogen atmosphere. The heating profile was ramped from ambient temperature to 70 °C at a rate of 10 °C ⋅ min^−1^, followed by a ramp from 70 °C to 400 °C at a rate of 1 °C ⋅ min^−1^, then from 400 °C to 1000 °C at a rate of 2 °C ⋅ min^−1^. The sample was maintained at 1000 °C for 15 min before being cooled down to ambient temperature. Finally, the sample was washed with 1 M HCl solution and H_2_O to dissolve the substrate MgO out, and dried at 105 °C for 24 h. The fabricated hard carbon samples denoted as BPPHC1000, as summarized in Figure 1.

### Materials characterization

The structure was characterized by an X′Pert Pro MPD X‐ray diffraction (XRD) (Philips, Netherlands) with CuKa radiation at 40 kV and 40 mA for 2 h in the range of 5–65°. The morphology of the hard carbon materials were characterized by SEM (JSM 6700, OLYMPUS, Tokyo, Japan) after coating with gold particles in a sputter coater Transmission electron microscopy (TEM) and high‐resolution transmission electron microscopy (HRTEM) images were taken with a JEOL n JEM 2011F apparatus operating at a voltage of 200 kV. The samples were dispersed in ethanol for 30 minutes before being transferred into the TEM chamber. They were then deposited onto a carbon‐coated copper grid, and then quickly moved into the vacuum evaporator. Nitrogen physisorption was used to determine the nitrogen adsorption and desorption isotherms on a Micrometritics ASAP 2020 analyzer.

### Electrochemical measurements

The preparation of the negative electrode involves several steps, including: slurry mixing, coating, drying, cutting, and weighing (to calculate the mass of the active material). First, the active material (prepared hard carbon material), conductive agent Ketjen Black, and binder PVDF are mixed in NMP at a certain mass ratio of 80 : 10 : 10, and stirred to prepare a homogeneous electrode slurry. The prepared slurry is coated on a copper foil and then scraped with a 200 μm scraper. The scraped electrode sheet is placed in a vacuum drying oven and dried at 80 °C under vacuum for 24 hours. The dried electrode sheet is cut into round electrode sheets with a diameter of 10 mm as the working electrode, and the active material loading of the electrode sheet is about 1.5 mg/cm^2^. All steps of the battery assembly are completed in a glove box, where the concentrations of H_2_O and O_2_ are kept below 0.01 ppm. The counter electrode and separator are made of suitable materials, such as metallic sodium and glass fiber membranes, respectively. The electrolyte used is 1 M NaPF_6_ with tetraethylene glycol dimethyl ether (DME). To assemble of the button cell, begin by preparing the negative electrode shell. Next, insert the sodium piece and add the electrolyte, separator, electrolyte again, hard carbon, gasket, spring and positive electrode shell. The amount of electrolyte used is 100 μL, which is added on both sides of the separator. To allow the electrolyte to fully wet, the assembled cell is left stationary at room temperature for 12 h before further electrochemical testing.

The capacitance performance of materials is tested and analyzed using the constant current charge‐discharge method. The assembled battery is installed on the LANDTE‐CT2001A battery testing system, and the battery is tested for charge‐discharge through the instrument. The testing voltage range is 0.01–2 V. The material‘s redox ability and cyclic characteristics are analyzed using Cyclic Voltammetry (CV). The assembled battery is installed on the PARSTAT P400A electrochemical workstation for testing. The battery is allowed to stand still for 12 hours before testing. The testing conditions are: scanning rate is 0.01–2 mV/s, 3 cycles, and voltage range is 0–2 V. Electrochemical Impedance Spectroscopy (EIS) is used to test and analyze the resistance behavior of materials in electrolytes. Typically, electrochemical impedance is tested on the PARSTAT P400A electrochemical workstation with a frequency range of 0.01–100 kHz.

## Supporting Information

Supporting Information is available from the Wiley Online Library or from the author.

## Conflict of interests

The authors declare no conflict of interest.

1

## Supporting information

As a service to our authors and readers, this journal provides supporting information supplied by the authors. Such materials are peer reviewed and may be re‐organized for online delivery, but are not copy‐edited or typeset. Technical support issues arising from supporting information (other than missing files) should be addressed to the authors.

Supporting Information

## Data Availability

The data that support the findings of this study are available in the supplementary material of this article.
