# Supplementary material for: Preparation of green high‐performance biomass‐derived hard carbon materials from bamboo powder waste
Source: ChemistryOpen. 2024 Jan 12;13(5):e202300178. doi: 10.1002/open.202300178 (PMC11095150; doi:10.1002/open.202300178)
Supplement: Supplementary file 1 — Supporting Information [file OPEN-13-e202300178-s001.pdf]

ChemistryOpen

Supporting Information

# Preparation of green high-performance biomass-derived hard carbon materials from bamboo powder waste

Tianqi Yin, Zhengli Zhang, Lizhi Xu, Chuang Li,* and Dongdong Han*

# Supplementary Information

**Contents**

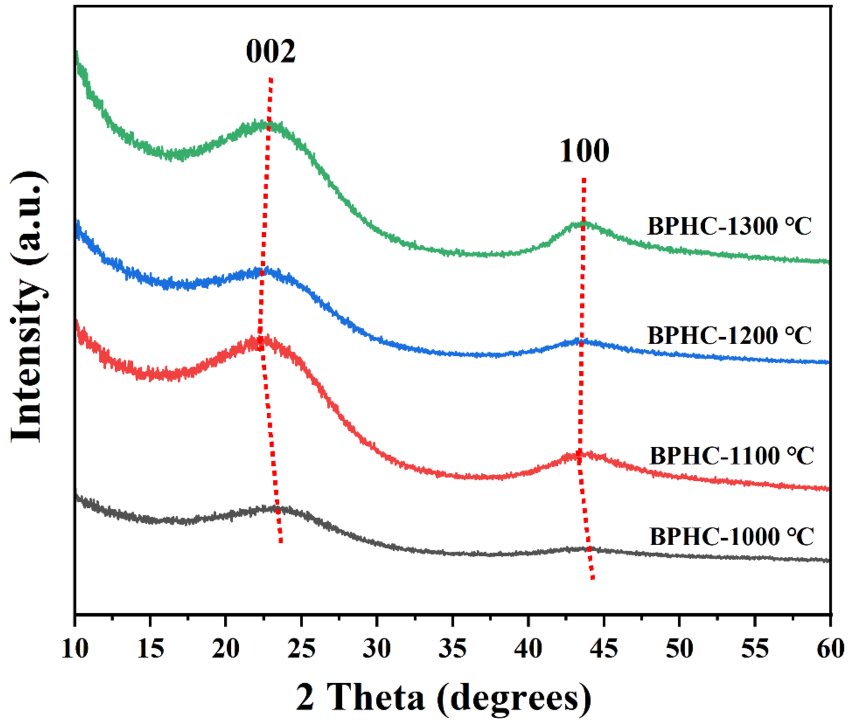


**Fig. S1** XRD patterns of different hard carbon samples: BPHC samples.

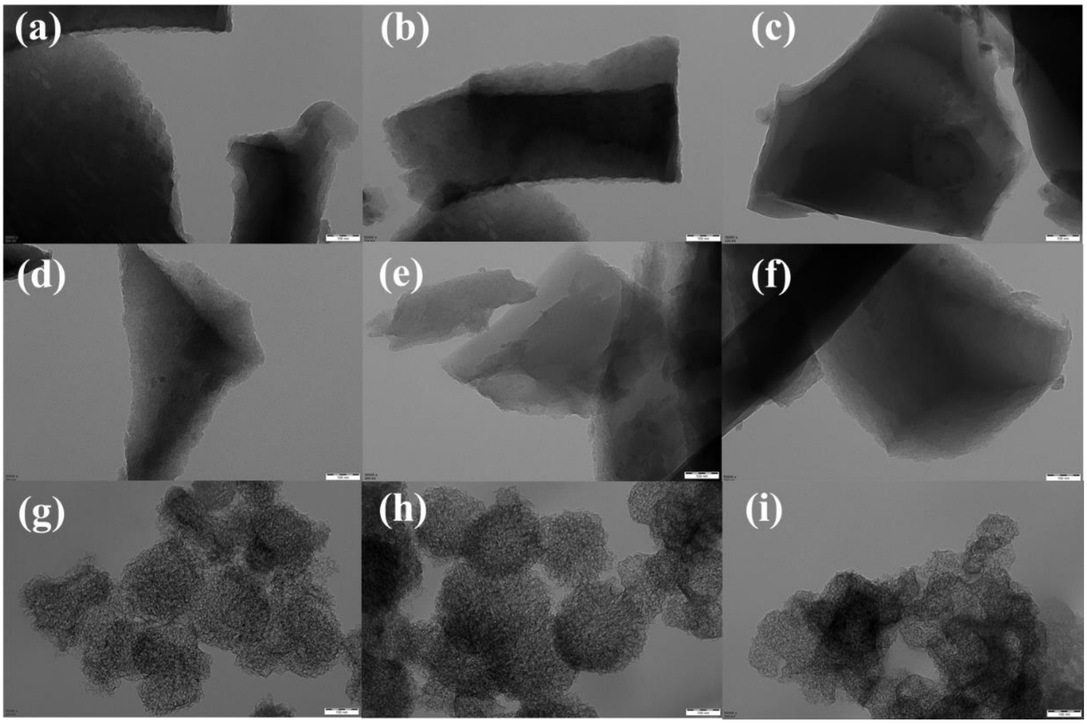


**Fig. S2** TEM images of (a) BHC1000, (b) BHC1100, (c) BHC1200, (d) BPHC1000, (e) BPHC1100, (f) BPHC1200, (g) BPPHC1000, (h) BPPHC1100 and (i) BPPHC1200.

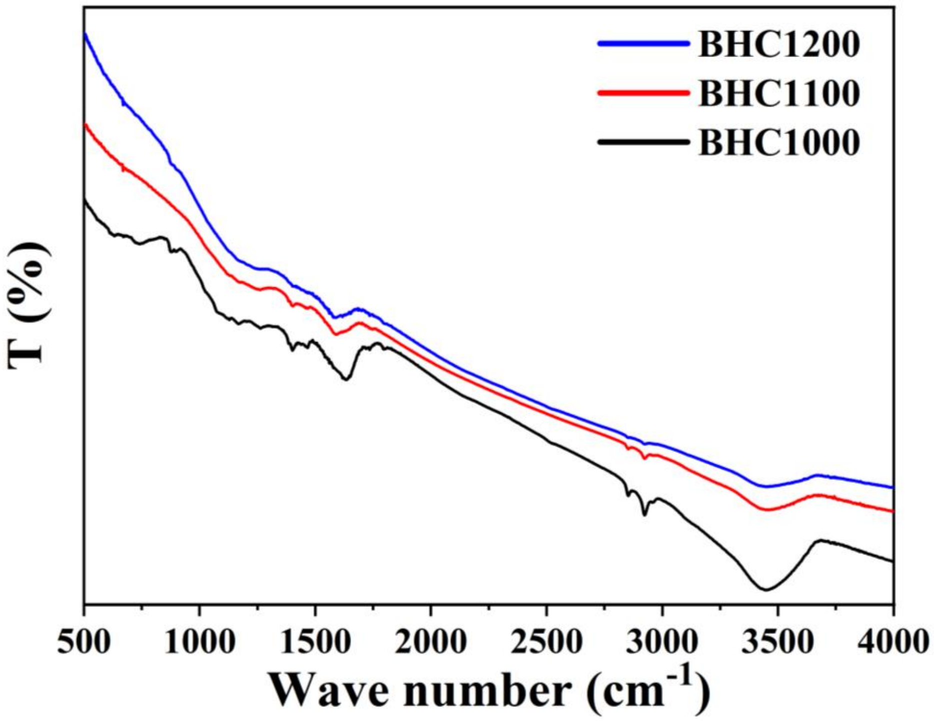


**Fig. S3** FTIR of BHC at temperatures of 1000, 1000, 1200

All samples have a characteristic peak at 3445 cm$^{-1}$, which corresponds to the -OH stretching vibration peak. The characteristic peaks of BHC1000 at 1736 cm$^{-1}$ and 1000~1200 cm$^{-1}$ correspond to the nonconjugate C=O stretching vibration peak and C-O stretching vibration peak, which indicates the presence of a high content of oxygen-containing functional groups.
